# Community Assessment for Mental and Physical Health Effects After Hurricane Irma — Florida Keys, May 2019

**DOI:** 10.15585/mmwr.mm7026a1

**Published:** 2021-07-02

**Authors:** Yaritbel Torres-Mendoza, Alison Kerr, Amy Helene Schnall, Carina Blackmore, Summer D. Hartley

**Affiliations:** ^1^Epidemic Intelligence Service CDC; ^2^Community Health Improvement and Planning, Florida Department of Health; ^3^National Center for Environmental Health, CDC; ^4^Division of Disease Control and Health Protection, Florida Department of Health; ^5^Health Affairs, West Virginia University, Charleston, West Virginia.

Disasters can adversely affect population health, resulting in increased need for health services. Hurricane Irma made landfall in the Florida Keys (Monroe County) as a Category 4 hurricane on September 10, 2017. The hurricane caused substantial damage to 65% of homes and resulted in 40 persons injured and 17 deaths from hurricane-related causes.* During 2018, the county suicide rate increased to 34.9 per 100,000 population from the 5-year (2013–2017) average of 25.2 per 100,000 population (*1*). In May 2019, 20 months after the hurricane, the Florida Department of Health (FDOH) conducted a modified Community Assessment for Public Health Emergency Response (CASPER) to assess the community’s mental, physical, and economic health and develop public health interventions to decrease the suicide rate. A consenting adult member from 231 households was interviewed, and a weighted cluster analysis was conducted to estimate the number and percentage of households throughout the Florida Keys with a particular response, as well as the number and percentage of persons at risk for suicide. During the 20 months since Hurricane Irma, 17% of households reported a need for a mental health care provider; 37.9% of these did not receive those services. A modified CASPER was used to calculate population estimates of suicide risk in an area of high landfall for hurricanes; estimated population suicide risk was 7.3%. Respondents reported worsening of respiratory conditions (17.7%), anxiety (17.0%), and depression (11.3%). Emergency preparedness plans should consider strengthening mental health service delivery after a hurricane, particularly during the long-term recovery phase. 

During May 21–23, 2019, FDOH conducted a modified CASPER in the Florida Keys (*2*). The sampling frame included all households in the Florida Keys according to the 2010 U.S. Census data. Census blocks were used as clusters to select nonoverlapping sections of the Florida Keys. To increase statistical power, FDOH selected 35 clusters rather than the traditional 30 (*2*). Interview teams comprising one FDOH staff member and one community member selected seven households from each cluster using systematic random sampling. Within households, teams randomly selected an adult aged ≥18 years.^†^ All participating households received brochures with information on health services provided locally and contact information for the suicide lifeline. If there was no answer after the first visit, teams left a written notification asking for a household member to call FDOH and provide their availability for completing the survey interview. Teams substituted households following the same systematic selection process after a home was found vacant, three visits were made with no answer, or a household member refused to participate. The goal was to complete 245 interviews in 3 days.

FDOH, along with state and local stakeholders, developed a questionnaire to assess mental, physical, and economic health. Suicide risk was assessed using the four-item Suicide Behaviors Questionnaire-Revised (SBQ-R) (*2*,*3*). Item one of the SBQ-R evaluates lifetime suicidal ideation and suicide attempt, item two assesses frequency of suicidal ideation during the past 12 months, item three evaluates lifetime threats of suicidal behavior, and item four assesses likelihood of suicidal behavior someday (*3*). The range of scores for each item are as follows: item one, 1–4; item two, 1–5; item three, 1–3; and item four, 0–6. A combined score of ≥7 indicates a lifetime risk for some suicide behavior with sensitivity of 93% and a specificity of 95% (*3*,*4*).

Respondents were asked about health conditions that had worsened, need for physical and mental health care, and barriers to receiving care among any household member since Hurricane Irma. Household-level weighted estimates, percentages, and 95% confidence intervals (CIs) were calculated based on the household’s selection probability. Individual-level weight was calculated to account for the probability of an adult being selected within the household for individual-level questions (e.g., SBQ-R). All weighted frequencies, percentages, and CIs were calculated in SAS software (version 1.7.0_76; SAS Institute).

This activity was reviewed by CDC and was conducted consistent with applicable federal law and CDC policy.^§^ The FDOH Ethics and Human Research Protection Program deemed the modified CASPER to be nonresearch with a primary intent of a public health response. All participants provided oral consent.

Field teams visited 458 households for potential survey inclusion. After accounting for replacements and refusals, field teams were able to enroll 231 households in the allotted time for a contact rate of 50.4% (the number of completed interviews divided by the total number of households for which contact was attempted). Eleven questionnaires were excluded from individual-level question estimates because of incomplete data that did not allow for individual weights. As a result, 220 questionnaires were available to evaluate individual-level responses. Median respondent age was 58 years (mean = 56 years; interquartile range = 44–67), 29% were aged ≥65 years, the median number of persons per household was two, and the median reported household income was $65,000. Seventeen percent of respondents reported that someone in the household needed mental health services. Among those, 37.9% did not receive needed services, with 56.2% reporting cost as a barrier (Table 1). Respondents were asked if “a person should generally sort out their own mental health problems”; 17.7% responded affirmatively. Thirteen questionnaires were excluded from the suicide risk estimate because at least one of the SBQ-R items was missing, for a final sample size of 207. The SBQ-R risk scores identified an estimated weighted suicide risk in the Florida Keys adult population of 7.3% (i.e., 7.3% are at some risk for suicide) (Table 1). 

**TABLE 1:** Estimated household mental health needs, barriers to care, and suicide risk 20 months after Hurricane Irma — Community Assessment for Public Health Emergency Response, Florida Keys, 2019

Characteristic	Household	
	Estimated no.	% (95% CI)
**Needed mental health services (n = 231)**		
Yes	7,657	17.0 (11.1–22.8)
No	35,104	77.7 (71.7–83.6)
Don’t know or refused	—*	—
Missing	1,639	3.6 (0.7–6.5)
**Received needed services (n = 38)^†^**		
Yes	4,651	60.7 (48.5–73.0)
No	2,899	37.9 (25.8–50.0)
Don’t know, refused, or missing	—	—
**Cost as a barrier to access care (n = 12)^§,¶^**		
Yes	1,629	56.2 (21.3–91.1)
No	1,270	43.8 (8.9–78.8)
Suicide risk, persons,** **SBQ-R Scale (n = 207)^††^**		
Risk^§§^	6,037	7.3 (3.3–11.2)
No risk	77,021	92.7 (88.8–96.7)

**Abbreviations:** CI = confidence interval; SBQ-R = Suicide Behaviors Questionnaire-Revised.

* Dashes indicate number of the responses was too few to be weighted for each option.

^†^ Only among those households responding that they needed mental health services.

^§^ Only among those households responding that they did not receive needed services.

^¶^ Multiple responses were permitted.

** Estimate based on the individual-level SBQ-R questions.

^††^ An adult is defined as being at risk if the SBQ-R score is ≥7.

^§§^ https://www.aetnabetterhealth.com/louisiana/assets/pdf/providers/communications/SDQ-Color.pdf

The three health conditions most commonly reported as worsening since the hurricane were respiratory problems (17.7%), anxiety (17.0%), and depression (11.3%) (Table 2). Sixty-four percent of households had at least one member who needed a primary care doctor or pediatrician for any health care need after the hurricane; 90.8% of those households received the needed care. Lack of doctors nearby and cost were the two barriers most frequently reported by persons who did not receive care.

**TABLE 2:** Estimated number and percentage of households reporting worsened health conditions, provider needs, and barriers to care 20 months after Hurricane Irma — Community Assessment for Public Health Emergency Response, Florida Keys, 2019

Characteristic	Household	
	Estimated no.	% (95% CI)
Worsened health conditions (n = 231)*		
Respiratory conditions	7,991	17.7 (13.0–22.4)
Anxiety	7,702	17.0 (10.1–23.9)
Depression	5,103	11.3 (5.7–16.9)
Fatigue	3,904	8.6 (3.7–13.6)
Insomnia	3,451	7.6 (4.0–11.3)
Hypertension	2,777	6.1 (2.0–10.3)
Other^†^	4,229	9.4 (4.5–14.2)
**Needed a primary care doctor or pediatrician (n = 231)**		
Yes	28,984	64.1 (55.9–72.4)
No	15,328	33.9 (26.1–41.8)
Don’t know, refused, or missing	—^§^	—
**Received needed services (n = 146)^¶^**		
Yes	26,328	90.8 (85.1–96.6)
No	2,433	8.4 (2.9–13.9)
Don’t know, refused, or missing	—	—
Barriers to access care (n = 12)**		
No doctor nearby	1,464	60.2 (25.5–94.8)
Cost	1,378	56.6 (23.3–90.0)

**Abbreviation:** CI = confidence interval.

* Multiple responses were permitted.

^†^ Includes diabetes, poor appetite, fibromyalgia, and other medical conditions.

^§^ Dashes indicate number of the responses was too few to be weighted for each option.

^¶^ Only among those households responding that they needed a primary doctor or pediatrician.

** Only among those households responding that they did not receive needed services.

## Discussion

The survey took place 20 months after Hurricane Irma in response to an increased suicide rate in the year after hurricane landfall. The modified CASPER aimed to assess the potential long-term effects of Hurricane Irma on the population. 

This study describes the use of a modified CASPER to establish a population estimate of suicide risk after a disaster. This assessment established a reference point for suicide risk in the Florida Keys after a hurricane using SBQ-R scores. The identified suicide risk will be compared with future suicide risk estimates for the evaluation of future mental health intervention strategies.

Although 17% of respondents reported a need for mental health services for at least one household member, approximately 40% of those needing such services did not receive them. The most commonly reported barrier to receiving mental health services was limited financial resources. Other reports have documented the increased need for mental health services after a disaster (*5*–*7*). The current findings highlight the importance of increasing access to mental health services after the initial phase of disaster relief, when assistance is readily available, specifically when communities realize the limitations of disaster assistance during the long-term recovery phase of a disaster (*6*).

Building mental health service delivery into emergency preparedness plans could help emergency management and public health programs address the mental health needs during short- and long-term disaster recovery, particularly among persons with limited financial resources. Stakeholders, including nonprofit organizations and mental health care providers, might consider increasing the visibility of mental health services when emotional lows are expected, such as during the next hurricane season and hurricane anniversaries (*6*). Primary care providers who are trained to recognize signs of depression and suicide risk can connect persons with needed services earlier. Suicide prevention needs can be addressed by incorporating prevention strategies from CDC’s Preventing Suicide: A Technical Package of Policy, Programs, and Practices (*8*).

In 64.1% of interviewed households, at least one resident needed a primary care doctor at some point during the 20 months after Hurricane Irma. Cost and unavailability of doctors were identified as barriers to accessing care. Providing transportation to health care services as part of disaster response, incentivizing providers to serve in person, and offering telemedicine services could increase access to care for underserved residents and those experiencing financial limitations (*9*,*10*). Access to health care services during a disaster response might also be improved by establishing connections with primary care and mental health service providers during the preparedness step of emergency management. 

The findings in this report are subject to at least three limitations. First, persons who were severely affected by the hurricane and relocated outside the Florida Keys were not captured in this assessment. Second, interview teams encountered many empty households, possibly because the Florida Keys is popular for seasonal residents and tourists, and the CASPER took place before seasonal residents were expected to return to their homes, which contributed to a low contact rate. Finally, because FDOH did not collect data on sex, race, and ethnicity, more detailed demographic information on respondents could not be reported. 

This modified CASPER was an important tool for evaluating the effects of Hurricane Irma by providing a point prevalence of the population’s suicide risk after a hurricane, identifying information gaps regarding community health needs, and providing information that will improve local disaster plans, response, and recovery activities. If resources allow, serial CASPERs could be conducted every 6 months throughout the duration of a hurricane or other environmental disaster recovery phases (immediate to long-term recovery) to address community needs in a timely manner. Emergency preparedness plans should consider strengthening mental health service delivery after a hurricane, particularly during the long-term recovery phase.

**Summary**

**What is already known about this topic?**

Community Assessment for Public Health Emergency Response (CASPER) is a useful tool to assess community needs after a disaster.

**What is added by this report?**

A modified CASPER was used to calculate population estimates of suicide risk in an area of high landfall for hurricanes; estimated suicide risk was 7.3%. During the 20 months after Hurricane Irma, residents of the Florida Keys reported worsening of anxiety (17.0%) and depression (11.3%) and a need for mental health services (17.0%).

**What are the implications for public health practice?**

Emergency preparedness plans should consider strengthening mental health service delivery after a hurricane or other disaster, particularly during the long-term recovery phase.

## Footnotes

*
https://www.monroecounty-fl.gov/726/Hurricane-Irma-Recovery

† An adult within a household was randomly chosen according to criteria determined by the interview team, such as the adult with the next birthday or the adult who correctly guessed a predetermined number.

§ 45 C.F.R. part 46, 21 C.F.R. part 56; 42 U.S.C. Sect. 241(d);5 U.S.C. Sect. 552a; 44 U.S.C. Sect. 3501 et seq.
